# Rare Fusion of the Semitendinosus and the Long Head of the Biceps Femoris Muscles in a Human Cadaver

**DOI:** 10.7759/cureus.12474

**Published:** 2021-01-04

**Authors:** Gabriella Schmuter, Sabastian Hajtovic, Rosalinda G Guce, Kiran Matthews

**Affiliations:** 1 Department of Anatomy, City University of New York (CUNY) School of Medicine, New York, USA

**Keywords:** hamstring muscle fusion, semitendinosus, hamstrings, fused muscles, biceps femoris

## Abstract

During routine cadaveric dissection of a 59-year-old female cadaver, a rare, anomalous fusion of the semitendinosus and long head of the biceps femoris muscles, arising as a common head at the origin of the ischial tuberosity, was observed. In addition, a unilateral muscular slip was noted between the gluteus maximus and the long head of the biceps femoris muscle belly. To the best of our knowledge, this variation has not been previously reported in the literature. Such variations may increase the risk of hamstring injury or compression of the sciatic nerve. Patients presenting with sciatic pain in the posterior thigh may prompt an evaluation for the aberrant origin of the hamstring muscles. It may be beneficial for surgeons, radiologists, and sports medicine specialists to be aware of such variations due to potential implications on surgical intervention, pain management, and interpretation of radiographic images.

## Introduction

Three muscles in the posterior compartment of the thigh comprise the “hamstrings”: the semimembranosus, semitendinosus, and long head of the biceps femoris (LBF), which are considered anatomically and functionally distinct. The LBF and the semitendinosus muscles classically originate as a conjoint tendon from the medial facet of the ischial tuberosity [1-3]. However, despite being tightly adjoined, their origins have been found to be identifiably distinct, with the semitendinosus having both a tendinous and muscular origin [4]. Both are involved in primary flexion of the knee joint and contribute to the extension of the hip joint.

Hamstring muscle injuries are very common in sports medicine [5-7], including acute strains, muscle rupture, avulsion of the proximal tendon origin, and chronic tendinopathy [7, 8]. Injury may be a result of the anatomic and functional features of the hamstring muscle complex [9]. The biceps femoris happens to be the most frequently injured hamstring muscle, perhaps due to its strong tendinous origin and consequent susceptibility to traction forces [4]. Knowledge of the location and severity of a hamstring strain is important for effective therapy, and for determining whether or not surgical intervention is indicated. Variations in hamstring structure may increase the risk of injury in affected individuals as a result of altered muscle glide and flexibility [10]. 

Aberrant muscular bundles between the LBF and the semitendinosus muscle have been reported [11-13]. However, to the best of our knowledge, fusion of the muscle heads has not been reported in the reviewed literature. Here, we present a rare case of fusion of the LBF and the semitendinosus muscles, with its common origin being at the ischial tuberosity.

## Case presentation

During routine dissection of a 59-year-old formalin-fixed female cadaver in the anatomy lab of the CUNY School of Medicine, a rare variation of the hamstring muscles was observed.

This 59-year-old female cadaver was registered with the Anatomical Gift Program at the University at Buffalo Jacobs School of Medicine. At the time of death, the body was fixed with formaldehyde injection into the femoral and internal jugular veins. The body was then transported to our institution and maintained with a 1% fabric softener solution to prevent dehydration and mold growth. No further preservation was necessary outside of the cranium.

Skin incision was made along the iliac crest: from the base of the sacrum to the anterior superior iliac spine; from the midline of the sacrum and coccyx to about 2 cm superior to the anus; and from the lower end of the previous incision, along the gluteal fold, to the lateral aspect of the gluteal region. Next, a midline incision in the posterior thigh was made from the gluteal fold to 4-5 cm below the popliteal fossa and extended laterally around the leg. Skin and superficial fascia were extended from the midline to the lateral aspect of the thigh, creating two flaps of skin that can be closed following dissection. After reflecting the gluteus maximus, the heads of the hamstrings in the posterior thigh were identified at their attachment to the ischial tuberosity. There was an attempt to separate the heads of the hamstring muscles by blunt dissection, which is when the semitendinosus and LBF muscles were found fused by a common head at the origin of the ischial tuberosity.

**Figure 1:**
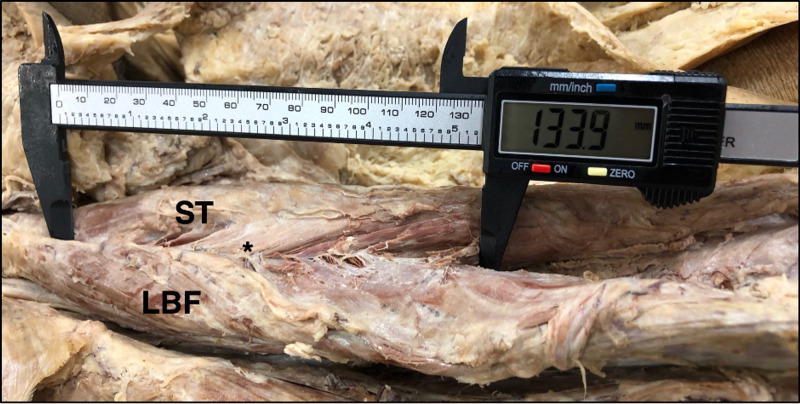
Length of Muscle Fusion Fusion of the semitendinosus (ST) and long head of the biceps femoris (LBF) indicated by the asterisk (*) measured as 133.9 mm in the posterior compartment of the left thigh

This rare anatomic fusion was found bilaterally. The full length of the fused muscle from the ischial tuberosity to the termination of the fusion was 133.9 mm in the left thigh (Figure 1) and 153.7 mm in the right thigh.

**Figure 2:**
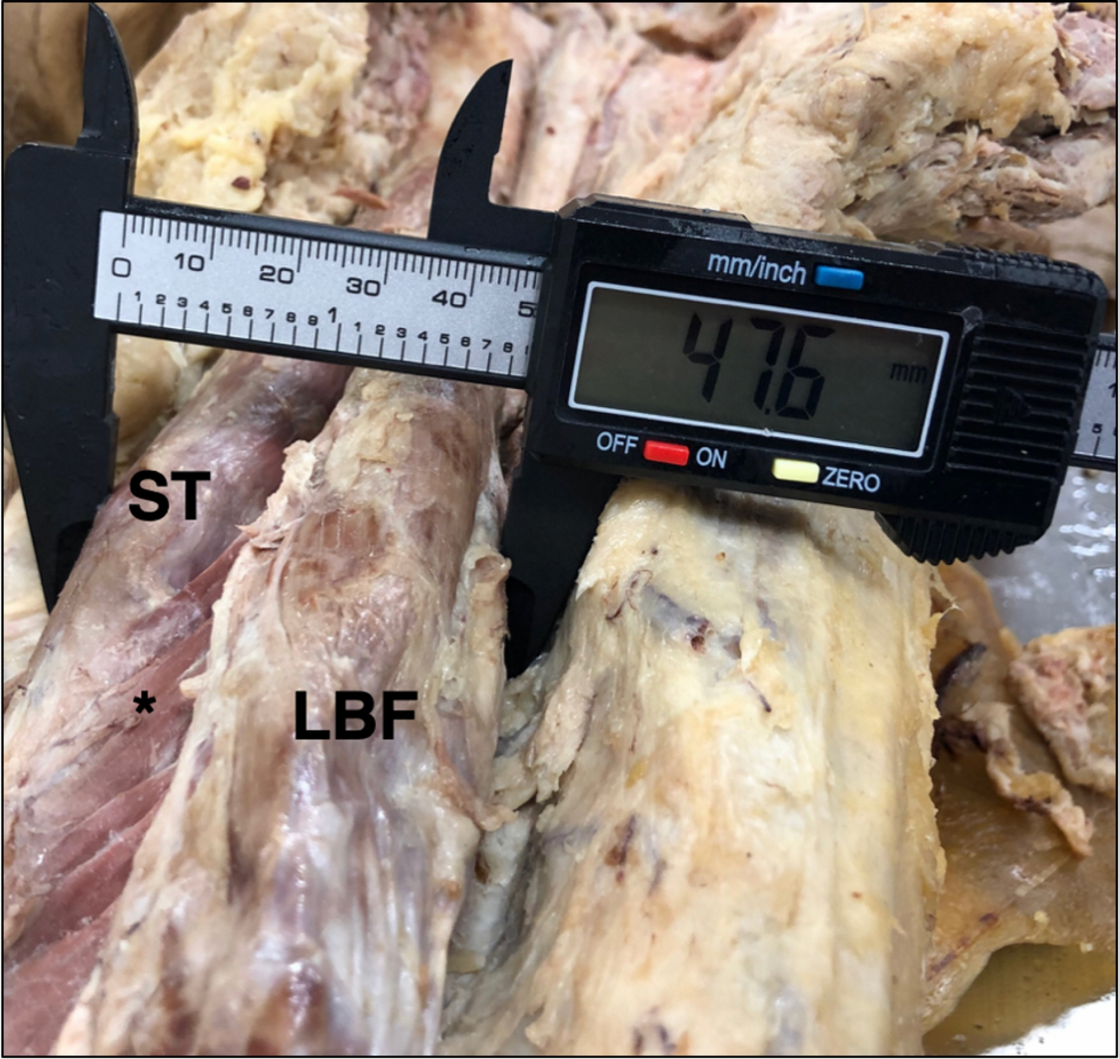
Width of Muscle Fusion Width of the muscular fusion (*) of the semitendinosus (ST) and long head of the biceps femoris (LBF), measured as 47.6 mm in the posterior compartment of the right thigh

At the widest point, the width of the fused muscle head was 52.9 mm and 47.6 mm (Figure 2) in the left and right thigh, respectively.

**Figure 3:**
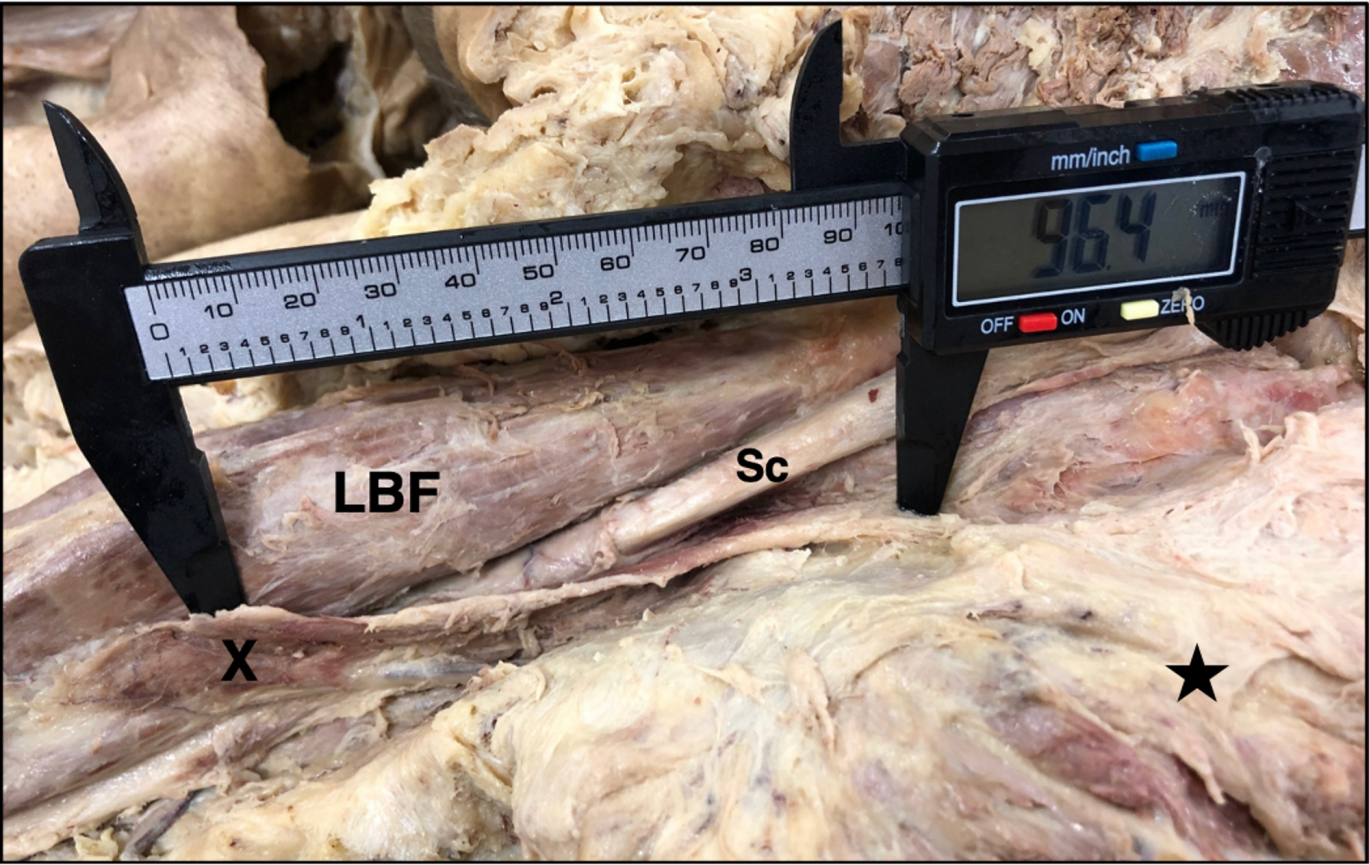
Aberrant Muscular Slip Muscular slip (X), arising from the lower border of the gluteus maximus muscle, reflected in this photo (star), and blending with the muscle belly of the long head of the biceps femoris (LBF) muscle. It measures 96.4 mm in the posterior compartment of the right thigh. The sciatic nerve (Sc) passes directly beneath the muscular slip, which is adjacent to the anomalous muscular fusion of the semitendinosus and LBF muscles

In the same cadaver, a muscular slip was found unilaterally on the right side, arising from the lower border of the gluteus maximus and blending with the fibers of the LBF. The length of the muscular slip from the gluteus maximus muscle to the point where it joins the LBF was 96.4 mm (Figure 3).

The muscular fusion between the semitendinosus and LBF on the right side was not altered by the presence of the muscular slip in the cadaver. No medical history pertinent to the lower extremity was documented in this cadaver. In addition, there were no signs of past surgical scars in the lower extremity.

## Discussion

Knowledge of hamstring variations may be relevant to general anatomical understanding. In addition, because both the semitendinosus and LBF are innervated by the tibial division of the sciatic nerve [1], consideration of possible variations in the posterior thigh may be useful in the management of certain neuropathies. This is particularly important in the case of sciatic nerve compression. The muscle bellies of the hamstring muscles are closely associated with the sciatic nerve; thus, injury to the hamstrings may result in simultaneous injury to the nerve bundle [14]. If a hamstring muscle variation were to increase the risk of acute strain or tendinopathy, sciatic nerve injury may also be more likely. Variations may predispose subsets of patients to hamstring injury by altering muscle glide and flexibility [10]. For instance, slips between hamstring muscles, as seen in our cadaver on the right side, may decrease flexibility due to adhesions [10]. Our cadaver, however, had no history of surgery or medical intervention in the lower limb. 

In this cadaver, at the widest point, the width of the fused muscles was 5.3 mm greater on the right side than the left, and 19.8 mm longer on the right side. It is therefore possible that the right thigh was at a greater risk of sciatic nerve compression, especially considering the presence of the anomalous muscular slip. The reason for this substantial difference in muscle length and width is unclear but may be related to increased use of the right lower limb during physical activity. 

Muscular variations of this nature may alter the biomechanics of the musculature. For instance, the presence of the muscular bundle between the LBF and the gluteus maximus muscle may strengthen both of these muscles to produce stronger hip extension. This may bring about compression of the sciatic nerve, resulting in a dragging pain in the lower limb while extending the hip. This biomechanical alteration may also occur from fusion of the two muscles.

In addition, an anomalous muscle originating from the LBF and merging with the semitendinosus has been reported [15], with the suggestion of such a muscle serving as a third head of the biceps femoris. Therefore, another possibility is that the muscular slip encountered in this cadaver could be serving this purpose as well.

## Conclusions

Fusion of the LBF and the semitendinosus muscle was observed near the origin at the ischial tuberosity, alongside a unilateral muscular slip, a variation that has not been previously documented in medical literature. Evaluation for such anatomic variations in patients may shed light on their effects on gait, pain, and other clinical parameters. Awareness of the potential for variation may be important for preventing complications of lower extremity neuropathies and management of hamstring repair. It is possible that such an anomaly may alter the course of surgical intervention or imaging evaluation of hamstring injuries.
